# Active surveillance of Q fever in human and animal population of Cyprus

**DOI:** 10.1186/1471-2334-6-48

**Published:** 2006-03-16

**Authors:** Fidias Loukaides, Christos Hadjichristodoulou, Elpidoforos S Soteriades, Virginia Kolonia, Maria-Christina Ioannidou, Anna Psaroulaki, Yannis Tselentis

**Affiliations:** 1 The Veterinary Services of Cyprus, Ministry of Agriculture, Athalassa avenue, Nicosia, Cyprus; 2 Department of hygiene and epidemiology, Medical Faculty, University of Thessaly, Papakyriazi 22, TK 41222, Larissa, Greece; 3 Laboratory of Clinical Bacteriology, Parasitology and Geographical Medicine, Faculty of Medicine, University of Crete, P.O. Box 1393, TK 71409, Heraklion, Crete, Greece

## Abstract

**Background:**

A long-term active surveillance of Q fever was conducted in Cyprus organized in two phases.

**Methods:**

Following serological tests and identification of seropositive humans and animals for *C. burnetii *in two villages (VIL1 and VIL2), all seronegative individuals were followed up for one year on a monthly basis by trained physicians to detect possible seroconversion for Q fever. In the second phase of the study, active surveillance for one year was conducted in the entire Cyprus. Physicians were following specific case definition criteria for Q fever. Standardized questionnaires, a geographical information system on a regional level, Immunofluorescence Assay (IFA) examinations and shell vial technique were used.

**Results:**

Eighty-one seronegative humans and 239 seronegative animals from both villages participated in the first phase surveillance period of Q fever. Despite the small number of confirmed clinical cases (2 humans and 1 goat), a significant percentage of new seropositives for *C. burnetii *(44.4% of human participants and 13.8% of animals) was detected at the end of the year. During the second phase of surveillance, 82 humans, 100 goats, and 76 sheep were considered suspected cases of Q fever. However, only 9 human, 8 goat, and 4 sheep cases were serologically confirmed, while *C. burnetii *was isolated from three human and two animal samples. The human incidence rate was estimated at 1.2 per 100,000 population per year.

**Conclusion:**

A small number of confirmed clinical cases of Q fever were observed despite the high seroprevalence for *C. burnetii *in human and animal population of Cyprus. Most of the cases in the local population of Cyprus appear to be subclinical. Moreover further studies should investigate the role of ticks in the epidemiology of Q fever and their relation to human seropositivity.

## Background

Q fever, a zoonosis distributed worldwide, is caused by *C. burnetii *and continues to be a public health problem in the entire Mediterranean region. *C. burnetii *is an obligate intracellular bacterium which lives in the phagolysosomes of the host cells and its most important hosts are ruminant animals (cows, sheep, goats) and domestic pets (cats, dogs, rabbits). *C. burnetii *has been associated with infertility and abortions in animals and is the causative agent of acute and chronic human cases of Q fever with considerable morbidity and mortality. Thus Q fever constitutes a public health problem with significant socioeconomic consequences [1-3].

Kaplan and Bertagna identified Q fever in Cyprus in 1951 for the first time [4]. During the years 1968 and 1969 Kelly examined 547 human blood samples from patients of Nicosia's General Hospital and 5% were found positive for *C. burnetii*. From December 1974 to June 1975, 78 British soldiers, who stationed in the Eastern Sovereign Base Area (ESBA) in Cyprus, contracted Q fever [5]. Epidemiological investigation revealed an abortion epidemic involving 21 mixed flocks of sheep and goats in the southeastern coastal region. Eleven of the flocks grazed in and around the ESBA. A serological survey of 10 affected flocks indicated that the abortion epidemic was the result of infection with *C. burnetii*. Transmission to humans was almost certainly acquired by inhalation of contaminated dust with *C. burnetii *after brushing birth products of the aborting animals. However, in the same region and during the same period no cases of Q fever were reported from the local population.

Before the current study an epidemiological study was conducted in Cyprus to estimate the seroprevalence for Q fever and identify "high risk" regions by using a computerized mapping program. The results of the above study are reported elsewhere. To further investigate the epidemiology of Q fever in Cyprus, we conducted the current study by establishing active surveillance of Q fever, to detect clinical cases in real time.

The main objectives of this study were to conduct active surveillance of Q fever, initially in "high risk" regions of Cyprus (VIL1 and VIL2) and afterwards in the entire human and animal population of Cyprus, to detect acute clinical cases of the disease and assess the pathogenicity of *C. burnetii*. The overall aim of this study was to assess if the small number of reported acute clinical cases of Q fever in the local population of Cyprus, despite the high seroprevalence (52.7%) identified in previous studies, is due to under diagnosis of Q fever or to under reporting of the disease.

## Methods

### Active surveillance of Q fever in the "high risk" region

Two villages of the rural area of Cyprus, VIL1 and VIL2, were determined as "high risk" regions (high seropositivity for *C. burnetii*) during the previous seroprevalence study. The entire population of both villages was invited to participate in the study (150 inhabitants from VIL1 and 198 from VIL2). The size of the random animal sample was calculated using an estimated 5% (± 3%) expected prevalence for sheep and goats, and 5% (± 4%) for bovine. All of the participants were initially serologically examined and all seropositive for *C. burnetii *were excluded from further follow up. Sera collected were examined for the detection of IgG, IgM, and IgA antibodies against *C. burnetii *phase II antigen by using indirect immunofluorescent antibody (IFA) test with a commercial kit (bioMerieux, Lyon, France). According to the test guidelines, IgG antibodies were accepted positive in case of > or = 1:60 titre, while IgM and IgA antibodies in case of > or = 1:25 titre [6].

The geographical information system was used on a regional level [7]. Information on risk factors was collected from the participants and the animal owners. The issues of the first phase were to detect clinical cases of Q fever, to isolate *C. burnetii *by shell vial technique and to assess the clinical spectrum of the disease in "high risk" region.

Following the detection of seropositive humans and animals in the "high risk" region, trained physicians followed all of the seronegative individuals up for one year on a monthly basis to detect clinical cases of Q fever. All the suspected cases of Q fever during the first year of follow up were examined serologically by IFA and attempts were made to isolate *C. burnetii *in confirmed cases. Detection of acute cases of Q fever was based on specific clinical and laboratory criteria. Acute *C. burnetii *infection was diagnosed on the basis of the association of (i) fever (38°C); with at least two other symptoms (chills, headache, myalgias, atypical pneumonia, and/or elevated hepatic transaminase levels) and with exhibition of a phase II immunoglobulin G (IgG) titre > = 1:240 and an IgM of > = 1:50 when only one serum specimen was available or a seroconversion when two sera sampled two or more weeks apart were available [8,9]. At the end of the follow up period, all participants were tested by IFA for identification of additional seroconversions even without overt clinical symptoms.

### Active surveillance of Q fever in the entire island of Cyprus

During the second phase of the study, an active surveillance of Q fever for one additional year in the whole human and animal population of Cyprus was conducted using the same methodology. All physicians in hospitals, health care centers and private medical facilities, in Cyprus, were asked to report suspected cases. As with the first phase, the total number of suspected cases was serologically examined by IFA and attempts were made to isolate *C. burnetii *via shell vial technique, in confirmed cases. Buffy coat inoculated on shell vials, which contained monolayers of Vero cells, and they were centrifuged at 700 × g for 1 h at 22°C. The inoculum was then removed and 1 ml of growth medium was added to the cells. The shell vials were incubated in a 5% CO2 incubator at 37°C. At the same time, two shell vials were inoculated with Minimal Essential Medium (MEM) and used as negative controls. The isolation of *C. burnetii *was carried out in a biosafety cabinet used only for shell vial infection. The cell monolayers in the shell vials were examined for *C. burnetii *by the IFA technique on day 6 and if the first test was negative, again on day 12 [10].

Active surveillance of Q fever in animal population of Cyprus was also conducted during the same study period. All the state and private veterinarians were asked to report to the veterinary services all the abortions and submit blood samples to be tested at the veterinary services laboratory. The IFA technique was used with a cut off point of IgG > = 1:240 and Igm> = 50. Moreover an effort was made to isolate *C. burnetii *via shell vial technique, in confirmed cases.

### Statistical analysis

Statistical analysis was performed using SPSS 11.0 and Epi-info 2002 software. We used chi-square for comparison of qualitative data and t-test or the non parametric mann-Whitney test for comparison of quantitative data.

## Results

### Active surveillance of Q fever in "high risk" region

Out of the total population in both villages under study, 79 individuals from VIL1 (response rate 52.46%) and 121 from VIL2 (response rate 61.8%) agreed to participate in the study. Fifteen participants from VIL1 (19%) and 21 from VIL2 (18%) were children. Ten percent of the animals owned by the participants were randomly sampled. Fifty five goats and 119 sheep were tested from VIL1 and 46 goats, 137 sheep and 26 bovine from VIL2.

The seropositivity of the inhabitants of **VIL1 **was estimated at 59.5% (47 individuals) using 1:60 titre as a cutoff point for IgG antibodies and at 52.9% (64 individuals) for the population of **VIL2**. The seropositivity was related to the participants' age. The mean age of seropositive participants was 44.7 years compared with 33.5 for seronegative and the difference was statistically significant (p < 0.05).

Seropositivity in children was 27.8% (10 out of the 36) and was significantly lower (RR = 0.46, p < 0.005) than the seropositivity of adults 61.6% (101 out of the 164). The animal seropositivity was correlated with the seropositivity of humans and was higher in **VIL1 (75/174, **43.1%) than the animal seropositivity in **VIL2 (60/209, **28.7%)using 1:60 titre as a cut off point for IgG antibodies (RR = 1.50, p < 0.005). The human seropositivity was related to animal contact (RR = 2.54, p < 0.0001). The relation was even stronger when the contact was with tick-infested animals (RR = 2.82, p < 0.0001).

Thirty two seronegative people for C. burnetii from VIL1 and 57 from VIL2 were followed up for one year on a monthly basis by trained physicians. Eight out of a total of 89 people were lost to follow up and finally 81 inhabitants actually participated in the follow up. Nineteen out of the 81 participants were children less than 15 years old. Thirteen suspected cases of Q fever were detected in both villages whereas only two cases were confirmed by laboratory testing (2.41%) representing an incidence rate of 5.7 per 1000 inhabitants of the specific region per year. Both cases detected in the "high risk" region were adults with mild symptoms treated as outpatients with favorable outcome.

Serological tests among the seronegatives at the end of the annual surveillance period revealed 36 new seropositive out of the 81 participants (44.4%) using 1/60 titre as a cutoff point for IgG antibodies while 20 residents (25.3%) were found positive for IgG antibodies using 1:120titre. Moreover, 19 participants (24.8%) were found positive for IgM antibodies using 1:25titre as a cutoff point. Six new seropositives were identified among the 19 (31.5%) participating children while 30 new seropositives were identified among the 62 (48.3%) participating adults (RR = 0.65, p = 0.30). The rate of acute Q fever cases versus the seropositives in the "high risk" region was estimated at 5.2% (2/38) using the 1:60 titre as a cutoff point and 9.09% (2/22) using the 1:120 titre as a cutoff point.

Forty five goats, 175 sheep and 19 bovine out of the total seronegative animal population in both villages were included in the active surveillance program. Although seven suspected cases of Q fever (3 goats and 4 sheep) were identified, from which only one goat was serologically confirmed, the percentage of new seropositive animals in the end of the year was 13.8% (33 new seropositive animals using 1:60 titre for IgG antibodies). The rate of acute Q fever animal cases versus the seropositive animals in the "high risk" region was estimated at 2.9% (1/34). In Table 1 the results from the annual surveillance period of Q fever in the two villages of Cyprus are presented.

**Table 1:** Seropositivity for *C. burnetii *in the "high risk" region of Cyprus before and after the surveillance period

	**Before**		**After**				
	Participants N	Seropositive n (%)	Seronegative participants n	Suspected cases n	Confirmed cases n	New IgG seropositive ≥ 1/120	New IgG seropositive ≥ 1/60

**Humans**	200	111 (55.5)	81*	13	2	20 (25.3)	36 (44.4)
**Animals**							
Goats	101	52 (51.4)	45	3	1	6 (13.3)	10 (22.2)
Sheep	256	65 (25.3)	175	4	-	17 (9.7)	21 (12)
Bovine	26	5 (19.2)	19	-	-	1 (5.2)	2 (10.5)
**Total **(animals)	383	122 (31.8)	239 **	7	1	24 (10)	33 (13.8)

### Active surveillance of Q fever in the entire island of Cyprus

The population of Cyprus is approximately 700,000 and the island has five major public hospitals in the capital of Nicosia and the other major cities of the island. The specific case definition criteria of Q fever, was distributed to all public hospitals, rural health care centers (approximately 30) and private health care facilities. Approximately, 200 primary care physicians were notified about the surveillance program. During one year of active surveillance in the entire island, 82 suspected cases were identified. Following laboratory examination, only nine cases were confirmed and *C. burnetii *was isolated from three human blood samples by shell vial technique. The incidence rate of Q fever in humans in Cyprus for this period of active surveillance was estimated at 1.2 per 100,000 inhabitants per year. The incidence rate in rural areas was estimated at 3.21 per 100,000 per year (7/218,000) and was higher of the estimated incidence rate in urban areas (0.41 per 100,000 per year, 2/482,000, RR = 7.74, p < 0.005). No sex difference was identified (5 males and 4 females). One out of the nine cases was a child l2 years old representing an incidence rate of 0.41 per 100,000 children per year while 8 cases were adults representing an incidence rate of 1.61 per 100000 adults per year (RR = 0.3, p = 0.4). Moreover all cases but one reported animal contact (goat or sheep).

The animal population of Cyprus during the study period was estimated at 374,000 animals (180,000 goats, 190,000 sheep and 4,000 bovines). One hundred goats and 76 sheep were reported to the Ministry of Agriculture as suspected cases of Q fever, however only 8 goats and 4 sheep were serologically confirmed during the one-year study period. *C. burnetii *was isolated from two goat blood samples by shell vial technique.

## Discussion

During an active surveillance program of Q fever in a "high risk" region of Cyprus (villages Kotsiatis and Paramali), among 81 seronegative participants who were followed-up on a monthly basis by trained physicians, thirteen individuals were clinically suspected for Q fever and only two of them were serologically confirmed as acute cases. Surprisingly, in the end of the year, 36 new IgG seropositive individuals for *C. burnetii *were detected (44.4%). If the seropositives together with the cases are regarded as exposed to *C. burnetii *then the estimated attack rate is 5.2% and the ratio among asymptomatic version symptomatic cases is 18, which is considered very high. The incidence rate (5.7/1000/year) in this "high risk" region was relatively high compared with other endemic regions worldwide [11-14] but it should be noted that this is a result of monthly follow up. Under the routine primary health care provision and surveillance, these two cases of Q fever with mild symptoms might not be diagnosed or reported. The estimated attack rate (5.2%) is very small compared with attack rates estimated during outbreak investigations in other endemic countries witch are reported up to 65% [11]. In the same region's animal population, among 239 seronegative animals, 7 suspected cases were identified and one was serologically confirmed. However, at the end of the year 33 new seropositive animals (13.8%) were detected. The attack rate among the animal population was estimated at 2.9%. The above findings supports that the exposure to *C. burnetii *in this specific region is high and that the majority of cases in the local population, appear to be subclinical. In a previous study in Cyprus *C. burnetii *was isolated in 7.8% of collected ticks by using shell vial technique [15]. Some authors suggest that in nature, *C. burnetii *is found primarily in a cycle involving ticks and free-living vertebrates and ticks play a significant role in the transmission of *C. burnetii *among the wild vertebrates, especially in rodents, lagomorphs, and wild birds. Other authors suggest that ticks transmit the agent not only to wild animals, but also to domestic animals (creating the livestock reservoir of *C. burnetii*) [1,16]. From our data and the previous study on ticks we could generate a hypothesis that ticks are related to seropositivity in both animals and humans but possibly not with human acute cases. This hypothesis should be further investigated by analytical epidemiological studies.

During an additional year of active surveillance in the entire island of Cyprus, only nine cases of Q fever human cases were serologically confirmed representing a relatively small incidence rate (1.2 per 100000 per year) compared to other endemic countries[11-14]. In Australia the incidence rate was estimated 30–60 cases per 100,000 per year in endemic regions while in Spain was estimated 5–10 cases per 100,000 per year. Comparing the incidence rate of Cyprus with other endemic countries we should take into consideration the fact that it was estimated after active surveillance. Both seropositivity and incidence rate of Q fever in Cyprus were lower in Children compared with adults. This finding supports the speculation of other researchers that children are less frequently symptomatic than adults following infection, and may have milder disease [17].

In endemic countries the diagnosis must be considered in the case of an unexplained fever, especially if the fever recurred following contact with possibly contaminated mammals. The best_tests _for diagnosis are those, which permit the direct detection of the bacteria. They include shell vial cell culture, PCR amplification, and immunodetection with tissue biopsy specimens. All these techniques require a level3 biosafety laboratory and trained personnel due to the extreme infectivity of *C. burnetii*. As for indirect specific diagnosis, the technique to be used should be very sensitive and should detect antibodies early in the course of the disease. In most of the countries serological diagnosis with IFA remains the only reliable alternative taking into consideration the problems with cutoff point values and the quality and standardization of the antigens [18,19].

Consequently, during the active surveillance for animal cases 12 animals were serologically confirmed and *C. burnetii *was isolated from one goat blood sample by shell vial technique. In the previous 6 years zero to one case were reported each year. This finding indicates that Q fever has been underreported in the past in the animal population of Cyprus either because the disease was not suspected or because there was no suitable laboratory network available.

It is important to point out that, unlike Cyprus, outbreaks of Q fever are often detected and investigated in other European countries where also high seroprevalence for *C. burnetii *is observed. In Vicenza (north eastern Italy), an outbreak of Q fever with 58 patients was detected, within a 5-month period, after exposure to three flocks of sheep that passed through the outbreak area [20]. Another large outbreak of Q fever, involving 415 serologically confirmed cases, was described in the Val de Bagnes (Switzerland) in 1983 [21]. In the island of Crete, in Greece, and in Marseille, France, 98 cases of acute Q fever over a fiveyear period, and 323 cases over an eight year period were identified, respectively [22,23].

Another observation from our study is that, in Cyprus as opposed to other countries, goats have the highest seropositivity for *C. burnetii *and are most often implicated in Q fever cases, whereas Q fever outbreaks are caused mainly from direct or indirect exposure to sheep flocks [24-27]. Moreover, although many Q fever cases, recently reported, were acquired from exposure to parturient cats [28,29], such correlation in Cyprus was not observed.

Our study suggests that due to the high prevalence of seropositivity in the human and animal population of Cyprus and the high proportion of subclinical cases in humans, active surveillance for the disease may be warranted in order to identify animal sources and control the transmission to the human population. Moreover, foreigners, who may not have immunity against *C. burnetii*, might be vulnerable to the disease as suggested by the level of seropositivity (2.7%) in Swedish soldiers serving in Cyprus in 1975 and 1976 [30] and the large number of Q fever cases in 1974 among British soldiers [5].

## Conclusion

The study shows that a large percentage of the population in Cyprus is seropositive to *C. burnetii *and that the majority of Q fever cases in the local population do not present with clinically overt symptoms. Therefore, people who get infected with *C. burnetii *are most often subclinical cases that neither are identified nor are reported. Moreover further studies should investigate the role of ticks in the epidemiology of Q fever and their relation to human seropositivity.

## Abbreviations

Immunofluorescence Assay (IFA)

Eastern Sovereign Base Area (ESBA)

Minimal Essential Medium (MEM)

## Competing interests

The author(s) declare that they have no competing interests.

## Authors' contributions

LF, HCh, and TY conceived and designed the study. LF, IMC and PA performed all the laboratory work. LF, HCh, SSE, KV collected blood samples and data. SSE drafted the manuscript and HCh reviewed it. All authors contributed to the final version of the manuscript, read and approved it.

## Pre-publication history

The pre-publication history for this paper can be accessed here:


